# Wear and Corrosion Resistance of Al_0.5_CoCrCuFeNi High-Entropy Alloy Coating Deposited on AZ91D Magnesium Alloy by Laser Cladding

**DOI:** 10.3390/e20120915

**Published:** 2018-11-30

**Authors:** Kaijin Huang, Lin Chen, Xin Lin, Haisong Huang, Shihao Tang, Feilong Du

**Affiliations:** 1 State Key Laboratory of Materials Processing and Die & Mould Technology, Huazhong University of Science and Technology, Wuhan 430074, China; 2 Material Corrosion and Protection Key Laboratory of Sichuan Province, Sichuan University of Science & Engineering, Zigong 643000, China; 3 State Key Laboratory of Solidification Processing, Northwestern Polytechnic University, Xi’an 710072, China; 4 Key Laboratory of Advanced Manufacturing Technology, Ministry of Education, Guizhou University, Guiyang 550025, China

**Keywords:** laser cladding, high-entropy alloy coating, AZ91D magnesium alloy, wear, corrosion

## Abstract

In order to improve the wear and corrosion resistance of an AZ91D magnesium alloy substrate, an Al_0.5_CoCrCuFeNi high-entropy alloy coating was successfully prepared on an AZ91D magnesium alloy surface by laser cladding using mixed elemental powders. Optical microscopy (OM), scanning electron microscopy (SEM), and X-ray diffraction were used to characterize the microstructure of the coating. The wear resistance and corrosion resistance of the coating were evaluated by dry sliding wear and potentiodynamic polarization curve test methods, respectively. The results show that the coating was composed of a simple FCC solid solution phase with a microhardness about 3.7 times higher than that of the AZ91D matrix and even higher than that of the same high-entropy alloy prepared by an arc melting method. The coating had better wear resistance than the AZ91D matrix, and the wear rate was about 2.5 times lower than that of the AZ91D matrix. Moreover, the main wear mechanisms of the coating and the AZ91D matrix were different. The former was abrasive wear and the latter was adhesive wear. The corrosion resistance of the coating was also better than that of the AZ91D matrix because the corrosion potential of the former was more positive and the corrosion current was smaller.

## 1. Introduction

High-entropy alloys are a new kind of alloy with excellent properties such as good wear resistance, excellent corrosion resistance, excellent oxidation resistance, low electrical conductivity, low thermal conductivity, and low coefficient of thermal expansion; they were invented by Yeh in 1995 [1,2,3,4,5,6,7]. They are composed of five or more elements in the same or an approximately equal molar ratio and have simple BCC and/or FCC solid solution phases. Their microstructure and properties are different from those of traditional alloys such as Fe- and Ni-based alloys or intermetallic compounds such as Ti–Al, Ni–Al, and Fe–Al compounds; this is because of the high entropy effect, severe lattice distortion effect, sluggish diffusion effect, and cocktail effect of the former [5,7].

So far, many methods such as arc melting [8], Tungsten Inert Gas Arc Welding (TIG) [9], Gas Tungsten Arc Welding (GTAW) [10], mechanical alloying [11], DC sputtering [12], thermal spay technology [13], and laser cladding [14,15,16,17,18,19,20,21,22,23,24,25,26,27,28,29,30,31,32] have been adopted to prepare different high-entropy alloys or coatings. Among them, the laser cladding method has attracted special attention because of its advantages of rapid heating, rapid cooling, compact coating, and low dilution rate, etc. [19]. At present, the preparation of high-entropy alloy coatings by laser cladding mainly focuses on the microstructure and properties of different matrix materials such as Ti-6Al-4V [14,15], carbon steel [16,17,18,19,20,21], stainless steel [22,23,24], tool steel [25], die steel [26], aluminum [27], magnesium [28,29,30], and magnesium alloys [31,32], and different high-entropy coatings [14,15,16,17,18,19,20,21,22,23,24,25,26,27,28,29,30,31,32]. It should be noted that the study of the preparation of high-entropy alloy coatings on magnesium and magnesium alloys by laser cladding was mainly carried out by a few researchers: Yue [28,29,31], Huang [32], and Meng [30]. The possible reason for this is that good-quality laser-clad coatings on magnesium and its alloys are very difficult to obtain because of its low melting point (922K) and low boiling point (1363K).

It is well known that magnesium and its alloys have been widely used in many fields such as the automotive, communication, and aerospace industries, but their poor wear resistance and corrosion resistance hinder their application in those situations where these properties are required. To solve this problem, a method called laser cladding has been developed to improve the wear and corrosion resistance of magnesium and its alloys, and numerous literature reports have confirmed this result [33,34]. Based on the excellent wear resistance and corrosion resistance of high-entropy alloys [1,2,3,4,5,6,7] and on the basis of an author’s previous research [32], we decided to continue the preparation of new wear-resistant and corrosion-resistant high-entropy alloy coatings by laser cladding on an AZ91D magnesium alloy substrate, which is widely used. To our best knowledge, no studies have reported an Al_0.5_CoCrCuFeNi high-entropy alloy coating fabricated by laser cladding on an AZ91D magnesium alloy substrate. The reason for studying the Al_0.5_CoCrCuFeNi alloy is that it has a simple FCC solid solution phase and exhibits excellent wear resistance due to its large work-hardening capacity [35].

In this paper, mixed powders of Cu, Ni, Al, Co, Cr, and Fe were used to fabricate an Al_0.5_CoCrCuFeNi high-entropy alloy coating on AZ91D magnesium alloys by laser cladding. The microstructure, wear behavior, and corrosion behavior of the coating are shown in detail.

## 2. Experimental

Laser cladding of mixed powders of Cu, Ni, Al, Co, Cr, and Fe was undertaken on an AZ91D magnesium alloy (Guangdong Dongguan Jubao Magnesium Alloy Material Co., Ltd, Dongguan, China). The sample size used in the laser cladding was 100 mm × 50 mm × 10 mm. The powders (Hebei Xingtai Nangong Zhongzhou Alloy Material Co., Ltd, Xingtai, China) had particle sizes of 48–75 μm and purity of 99 wt %. The different element powders were mixed by ball milling (DECO-PBM-V-0.4L, Hunan Changsha Deco Instrument Equipment Co., Ltd, Changsha, China) according to the nominal composition of the Al_0.5_CoCrCuFeNi high-entropy alloy. The mixed powder from the ball milling was preset on the surface of the AZ91D magnesium alloy using 4 vol % PVA solution. The thickness of the preset layer was about 0.6 mm. The laser cladding experiment was completed under the protection of 99.999% high-purity argon by using a TR050 type CO_2_ high-power laser. The optimized laser cladding parameters were as follows: laser power was 3000 W, laser scanning speed was 10 mm/s, laser spot diameter was 4 mm, and laser spot overlap rate was 25%.

The phase structure and microstructure of the coating after laser cladding were identified and observed using an X-ray diffractometer (X’ Pert PRO), an optical microscope (Axiovert 200MAT), and a scanning microscope (Quanta 400) with an energy spectrum, respectively. The microhardness of the coating cross section was measured using a microhardness tester (MICROMET 3). The loading force was 100 grams and the loading time was 15 s.

**Figure 1:**
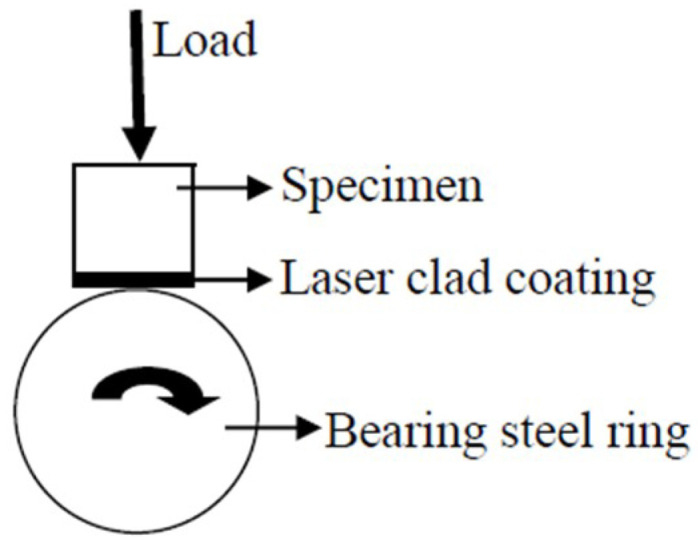
Schematic illustration of the block-on-ring sliding wear tester.

The wear resistance of the coating was evaluated by the dry sliding wear method, in which the size of the sample used for testing was 10 mm × 10 mm × 10 mm, and the friction pair used for matching was bearing steel (AISI52100, HV_0.1_700). Figure 1 shows a schematic illustration of the block-on-ring sliding wear tester. The parameters for the dry sliding wear were as follows: the applied load was 98 N, the dry sliding speed was 0.4187 m/s, the dry sliding wear time was 75 mins, and the dry sliding wear distance was 1884 m. The AZ91D magnesium alloy was selected as the experimental material for the dry sliding wear comparison. For each experimental datapoint, the average value of three experimental results was taken as the final data. Wear weight loss was measured using an electronic analytical balance (Bartorius BS110) with an accuracy of 0.1 mg.

The corrosion resistance of the coating was evaluated by testing the corrosion potential polarization curve of a 3.5 wt % sodium chloride solution. A saturated calomel electrode (SCE) was used as the reference electrode and platinum was used as the counter electrode. The test was performed from −2.5 V to 1.5 V with a scanning speed of 1 mV/s.

The surface morphologies of the worn and corroded specimens were observed using a scanning microscope (Quanta 400) with an energy spectrum.

## 3. Results and Discussion

### 3.1. Microstructure and Microhardness

**Figure 2:**
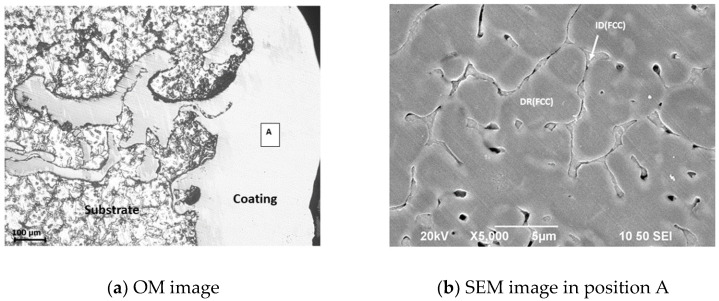
Morphology of a laser-clad Al_0.5_CoCrCuFeNi high-entropy alloy coating: (**a**) optical microscope (OM) image; (**b**) SEM image in position A.

Figure 2a shows an optical image of the cross section of the coating/AZ91D substrate sample after laser cladding. It can be seen from Figure 2a that there were basically no defects such as microcracks or pores in the coating. The interface between coating and substrate indicates that they were well combined. In addition, small pieces of AZ91D magnesium alloy substrate were also found in the coating (Figure 2a). This is believed to be due to some partially melted Mg being detached from the substrate and becoming trapped within the rapidly solidifying coating. Figure 2b shows the scanning electron microscopy morphology of position A in Figure 2a. It can be seen from Figure 2b that the coating has typical dendrite microstructure characteristics, and the chemical compositions of the dendrite (DR) and inter-dendrite (ID) are given in Table 1. As shown in Table 1, copper segregates significantly in the inter-dendrite region. In other words, local segregation of copper occurred in the Al_0.5_CoCrCuFeNi high-entropy alloy coating prepared by laser cladding. These results are similar to the microstructure characteristics of Al_0.5_CoCrCuFeNi high-entropy alloys prepared by arc melting in the literature [35,36,37,38]. The difference is that the dendrite microstructure of the laser cladding coating is fine due to the rapid heating, melting, and rapid solidification of the preset layer during laser cladding.

The copper segregation in the Al_0.5_CoCrCuFeNi high-entropy alloy can be explained by the mixing enthalpies [39] of different atomic pairs in Table 2. As shown in Table 2, all of the mixing enthalpies of copper with iron, chromium, cobalt, and nickel are positive. This means that copper has a low affinity for these atoms and is easily repelled by them. In the process of laser cladding, different elements in the Al_0.5_CoCrCuFeNi high-entropy alloy were mixed evenly due to the convection and agitation of the laser cladding pool. However, during the cooling and solidification stage, copper was excluded from the dendrites due to the low affinity of copper atoms with iron, chromium, cobalt, and nickel; thus, copper segregation occurred at the inter-dendrites.

**Figure 3:**
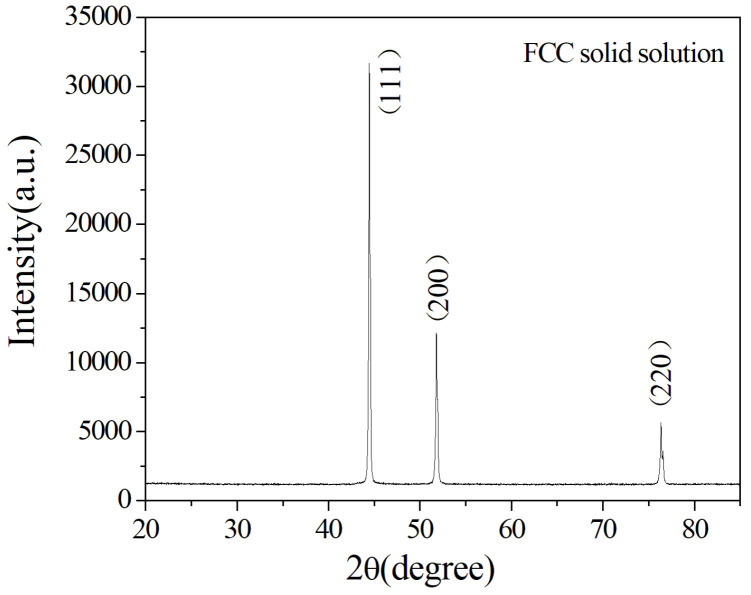
XRD patterns of a laser-clad Al_0.5_CoCrCuFeNi high-entropy alloy coating.

Figure 3 presents the XRD patterns of the Al_0.5_CoCrCuFeNi high-entropy alloy coating prepared by laser cladding. The analytical result confirmed that the phase was a simple FCC solid solution in the laser-clad coating. This result proved that the Al_0.5_CoCrCuFeNi high-entropy alloy coating was successfully fabricated by laser cladding on the AZ91D magnesium alloy based on the mixed powders of Cu, Ni, Al, Co, Cr, and Fe of Al_0.5_CoCrCuFeNi and the optimized laser cladding parameters. This result is consistent with the results reported in the literature [35,36,37,38]. Thus, for the laser-clad Al_0.5_CoCrCuFeNi high-entropy alloy coating, both the dendrites and Cu-rich inter-dendrites were of one simple FCC phase (Figure 1b). The formation of a simple solid solution phase rather than intermetallic compounds is mainly attributed to the significant lowering of free energy by the high enthalpy of mixing [40].

**Figure 4:**
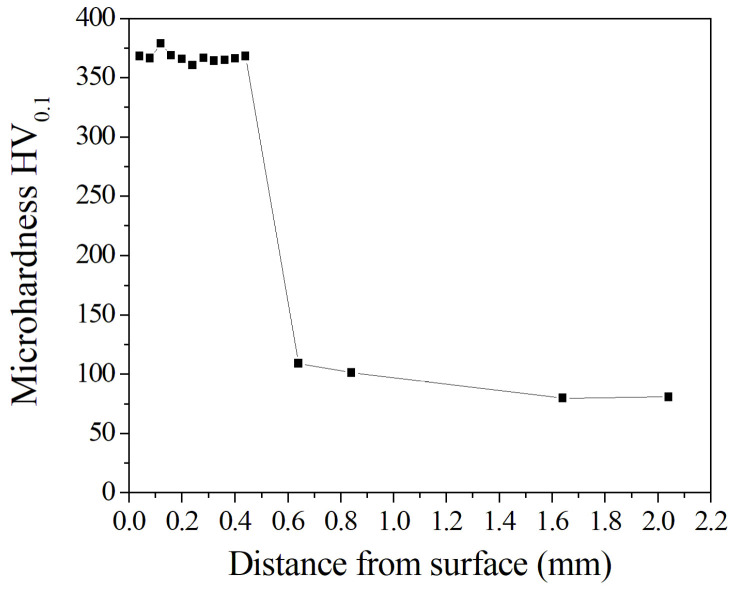
Microhardness of a laser-clad Al_0.5_CoCrCuFeNi high-entropy alloy coating.

Figure 4 shows the microhardness of the laser-clad Al_0.5_CoCrCuFeNi high-entropy alloy coating. As can be seen from Figure 4, the microhardness of the coating was HV_0.1_365—about 3.7 times of the AZ91D matrix (HV_0.1_98) and higher than that of the Al_0.5_CoCrCuFeNi high-entropy alloy (HV_5_233) prepared by arc melting [35,36,37]. The high microhardness of the laser-clad coating was attributed to the solid solution strengthening of different alloy elements and fine grain strengthening of the fine dendritic structure during the cooling process. Obviously, the high microhardness of the coating is more beneficial to increasing the wear resistance of the coating.

### 3.2. Wear Properties

**Figure 5:**
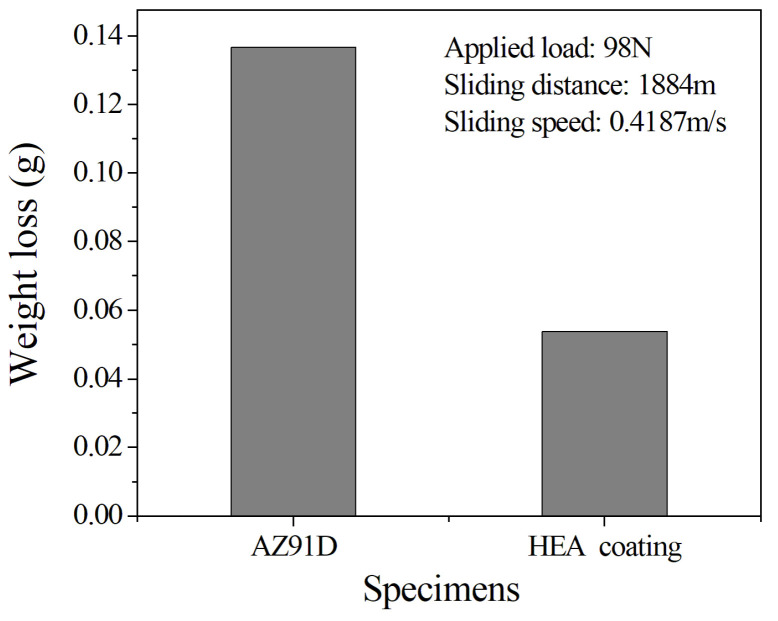
Weight loss of different specimens.

**Figure 6:**
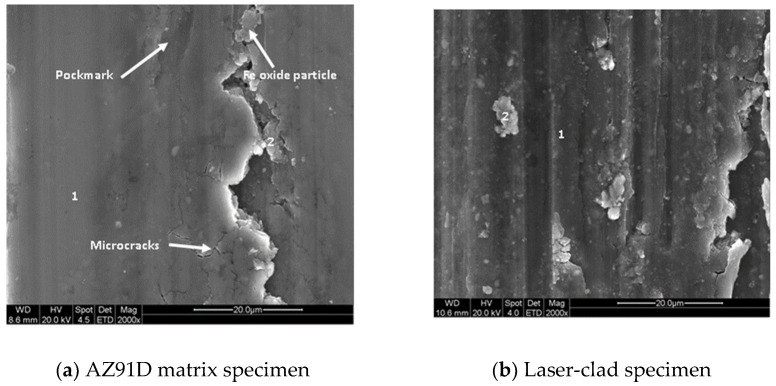
Worn surfaces of (**a**) an AZ91D matrix specimen and (**b**) a laser-clad specimen.

Figure 5 shows the dry sliding wear weight loss of different samples. According to Figure 5, the wear resistance of the coating was better than that of the AZ91D matrix, and the wear weight loss was about 2.5 times smaller than that of the AZ91D matrix. This is in accordance with their corresponding microhardness values (Figure 4). In other words, the hardness is higher and the wearability is better. Figure 6 shows the surface morphologies of different samples after dry sliding wear. In Figure 6, both of them present obvious groove characteristics. However, the wear mechanisms of the two samples were also significantly different. The coating showed obvious abrasive wear characteristics, and the AZ91D matrix showed obvious adhesive wear characteristics. Some elongated pockmarks and microcracks (Figure 6a) can be found on the worn surface of the AZ91D matrix. Table 3 shows the EDS results of different positions of different worn samples. It can be seen from Table 3 that AZ91D matrix position 1 and coating position 1 are each matrix materials, although each has a small amount of oxidation due to friction heating. Both AZ91D matrix position 2 and coating position 2 are iron oxide particles, but there are a lot of fine particles on the worn surface of the coating, while the worn surface of the AZ91D matrix is much cleaner.

The causes of the above phenomena should be related to the hardness of the respective matrix materials. Figure 4 shows that the microhardness of the coating was about HV_0.1_365, while the microhardness of the AZ91D matrix was only HV_0.1_98. In addition, the coating is composed of Al_0.5_CoCrCuFeNi high-entropy alloy, and Al_0.5_CoCrCuFeNi high-entropy alloy has superior work-hardening characteristics [35], but the AZ91D matrix does not have the same excellent work-hardening capability. As a result, the microhardness of the coating continued to increase when dry sliding wear was applied to the bearing steel friction pair and may even approach or exceed the hardness of the bearing steel friction pair; it would then shear off the steel ring material, and iron wear debris would be smeared over the coating surface. With the extension of the dry sliding wear time, the iron wear debris would be oxidized due to heat generated by friction. Therefore, iron oxide abrasive particles will be used as an abrasive cutting the Al_0.5_CoCrCuFeNi high-entropy alloy coating. In contrast, the AZ91D matrix did not have this process and only the soft AZ91D matrix was transferred to the hard bearing steel surface, so its wear rate (30.38 μg/s) was much higher than that (11.93 μg/s) of the coating.

### 3.3. Corrosion Properties

**Figure 7:**
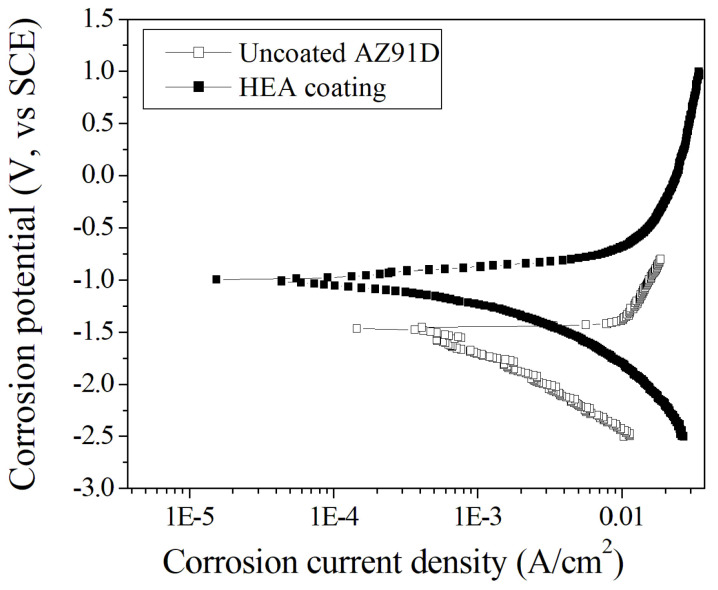
Potentiodynamic polarization curves of different specimens in 3.5 wt % sodium chloride solution.

Figure 7 and Table 4 show the potentiodynamic polarization curves and corrosion properties, respectively, of different samples in 3.5 wt % sodium chloride solution. According to the corrosion electrochemical behavior of the two samples and the lack of a passivating region in Figure 7, both the AZ91D matrix and the coating are active dissolved materials. Therefore, using the principle of “the smaller the corrosion current and the higher the corrosion potential, the better the corrosion resistance of the material” to evaluate the corrosion resistance of the active dissolved material, it can be seen that the corrosion resistance of the Al_0.5_CoCrCuFeNi high-entropy alloy coating prepared by laser cladding was better than that of the AZ91D matrix. This is reflected by the fact that the former had a significantly higher corrosion potential (E_corr_ = −0.998 V) than did the latter (E_corr_ = −1.46 V), and the former also exhibited a lower corrosion current (i_corr_ = 1.60 × 10^−4^ A/cm^2^) than did the latter (i_corr_ = 6.20 × 10^−4^ A/cm^2^).

**Figure 8:**
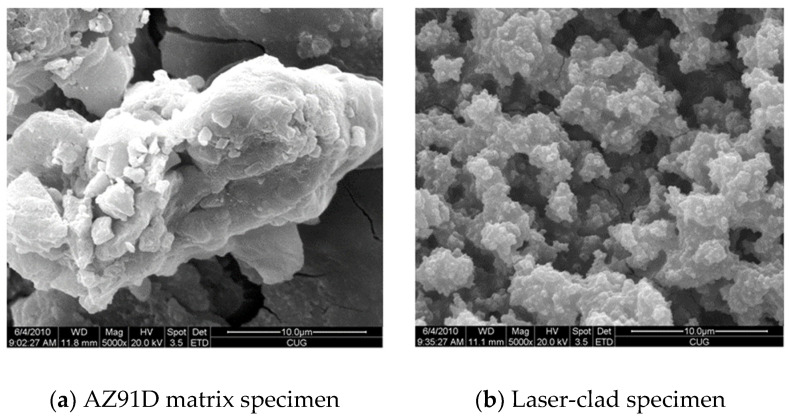
Corroded surfaces of (**a**) an AZ91D matrix specimen and (**b**) a laser-clad specimen.

Figure 8 shows the corroded surfaces of two samples. It can be seen from Figure 8a that the magnesium oxide film generated on the AZ91D matrix cannot provide effective protection due to its loose and porous properties and the extremely low equilibrium potential (−2.37 V) of magnesium in the AZ91D matrix (Table 3). The following reactions will occur [41]:

Mg(s) + H_2_O → Mg(OH)_2_(s) + H_2_(g),

Mg(OH)_2_(s) + Cl^−^ → MgCl_2_ + OH^−^,

Mg(s) + Cl^−^ (aq) → MgCl_2_.

Therefore, the corrosion of the AZ91D matrix was severe (Table 4 and Figure 8a).

On the other hand, the corrosion attack on the Al_0.5_CoCrCuFeNi high-entropy alloy coating specimen was much less acute. Figure 8b reveals that the corroded surface has a grainy appearance, indicating that corrosion attacks may mainly occur on dendrite boundaries. It is considered that the presence of Cr, Al, and Ni in the Al_0.5_CoCrCuFeNi high-entropy alloy coating prepared by laser cladding could form dense chromium oxides, aluminum oxides, and nickel oxides at the surface, and this could be an important factor that contributes to the relatively high corrosion resistance of the Al_0.5_CoCrCuFeNi high-entropy alloy coating specimen.

It should be pointed out that the composition of the high-entropy Al_0.5_CoCrCuFeNi selected in this paper is not superior to that of other high-entropy compositions (for example, the one studied in Reference [32]). In this paper, only the high-entropy Al_0.5_CoCrCuFeNi composition is chosen because of its good corrosion resistance.

As for how to choose the composition of the coating to obtain high corrosion resistance, we think that the composition of the high-entropy alloy should be based mainly on whether the same high-entropy alloy prepared by the arc melting method has excellent corrosion performance. If so, it can be selected as a candidate material for laser cladding to prepare a high-entropy alloy coating; otherwise, it should not be selected.

## 4. Conclusions

(1). An Al_0.5_CoCrCuFeNi high-entropy alloy coating was successfully fabricated on an AZ91D matrix by laser cladding using mixed powders of Cu, Ni, Al, Co, Cr, and Fe. The Al_0.5_CoCrCuFeNi high-entropy alloy coating consisted of fine dendrites with a simple FCC phase.
(2). The dry sliding wear resistance of the Al_0.5_CoCrCuFeNi high-entropy alloy coating prepared by laser cladding was better than that of the AZ91D matrix, and the wear mechanisms of the two materials were different.
(3). The Al_0.5_CoCrCuFeNi high-entropy alloy coating prepared by laser cladding had better corrosion resistance than did the AZ91D matrix in 3.5 wt % sodium chloride solution.

## Figures and Tables

**Table 1:** EDS results of the laser-clad high-entropy alloy coating (atom %).

Elements	Al	Co	Cr	Cu	Fe	Ni	Results
DR	9.9	18.7	20.1	12.1	22.0	17.2	poor in Cu element
ID	12.7	5.7	3.5	60.7	3.1	14.3	rich in Cu element
Raw powders	9.0	18.2	18.2	18.2	18.2	18.2	

**Table 2:** Values of ΔH_ij_^mix^ (kJ/mol) for atomic pairs of elements [39].

Mixing Enthalpy	Al	Co	Cr	Cu	Fe	Ni
Al	-	−19	−10	−1	−11	−22
Co		-	−4	6	−1	0
Cr			-	12	−1	−7
Cu				-	13	4
Fe					-	−2
Ni						-

**Table 3:** EDS results of worn surfaces of the AZ91D matrix and the laser-clad specimen (wt %).

Samples	Position	C	O	Mg	Al	Zn	Fe	Ni	Cu	Cr	Co	Mn
AZ91D	1	2.2	6.5	83.8	6.8	0.7	-	-	-	-	-	-
AZ91D	2	1.8	44.5	1.3	1.3	0.3	50.8	-	-	-	-	-
Coating	1	1.1	8.9	-	4.7	-	18.7	19.9	12.4	17.3	17.0	-
Coating	2	1.2	33.7	-	3.4	-	34.8	5.3	7.2	7.1	7.3	-
AZ91D	Raw	-	-	90.2	8.9	0.6	-	-	-	-	-	0.3

**Table 4:** Corrosion parameters of different samples in 3.5 wt % sodium chloride solution.

Specimen	E_corr_ (V)	I_corr_ (A/cm^2^)
AZ91D	−1.46	6.20×10^−4^
Laser-clad coating	−0.998	1.60×10^−4^
